# Intra- and interspecies gene expression models for predicting drug response in canine osteosarcoma

**DOI:** 10.1186/s12859-016-0942-8

**Published:** 2016-02-19

**Authors:** Jared S. Fowles, Kristen C. Brown, Ann M. Hess, Dawn L. Duval, Daniel L. Gustafson

**Affiliations:** Cell and Molecular Biology Program, Department of Clinical Sciences, Colorado State University, Fort Collins, CO USA; Flint Animal Cancer Center, Veterinary Medical Center, Colorado State University, Fort Collins, CO USA; Cell and Molecular Biology Program, Department of Biology, Colorado State University, Fort Collins, CO USA; Department of Statistics, Colorado State University, Fort Collins, CO USA; Shipley University Chair in Comparative Oncology, Flint Animal Cancer Center, Room 246, Colorado State University VMC, 300 West Drake Road, Fort Collins, CO 80523-1620 USA

**Keywords:** Osteosarcoma, Canine, Human, COXEN, Gene expression modeling

## Abstract

**Background:**

Genomics-based predictors of drug response have the potential to improve outcomes associated with cancer therapy. Osteosarcoma (OS), the most common primary bone cancer in dogs, is commonly treated with adjuvant doxorubicin or carboplatin following amputation of the affected limb. We evaluated the use of gene-expression based models built in an intra- or interspecies manner to predict chemosensitivity and treatment outcome in canine OS. Models were built and evaluated using microarray gene expression and drug sensitivity data from human and canine cancer cell lines, and canine OS tumor datasets. The “COXEN” method was utilized to filter gene signatures between human and dog datasets based on strong co-expression patterns. Models were built using linear discriminant analysis via the misclassification penalized posterior algorithm.

**Results:**

The best doxorubicin model involved genes identified in human lines that were co-expressed and trained on canine OS tumor data, which accurately predicted clinical outcome in 73 % of dogs (*p* = 0.0262, binomial). The best carboplatin model utilized canine lines for gene identification and model training, with canine OS tumor data for co-expression. Dogs whose treatment matched our predictions had significantly better clinical outcomes than those that didn’t (*p* = 0.0006, Log Rank), and this predictor significantly associated with longer disease free intervals in a Cox multivariate analysis (hazard ratio = 0.3102, *p* = 0.0124).

**Conclusions:**

Our data show that intra- and interspecies gene expression models can successfully predict response in canine OS, which may improve outcome in dogs and serve as pre-clinical validation for similar methods in human cancer research.

**Electronic supplementary material:**

The online version of this article (doi:10.1186/s12859-016-0942-8) contains supplementary material, which is available to authorized users.

## Background

Recent breakthroughs in cancer genomics have made the emerging field of personalized medicine not only a plausible but effective alternative to traditional approaches to cancer treatment. The discovery of biomarkers such as the breakpoint cluster region/Abelson (BCR/ABL) gene in patients with chronic myeloid leukemia, the Estrogen Receptor (ER) status of breast cancer patients, and the v-Raf murine sarcoma viral oncogene homologue B1 (BRAF) mutational status of melanoma patients has resulted in the development of effective targeted agents and successful patient stratification leading to better clinical outcomes [1]. In the case of ER and BRAF, their expression has been found to be prognostic in other tumor types as well, such as ER for non-small-cell lung cancer and the BRAF V600E mutant being observed in 45 % of papillary thyroid cancers and 11 % of colorectal cancers, showing the potential for predictive molecular characteristics to be extrapolated across histological boundaries [2–5]. Although success of single biomarkers in predicting response to molecularly targeted agents has been observed, this is not the case with traditional chemotherapeutics, which are still heavily used in human and veterinary medicine. Typically a host of genes involved in processes such as drug activation, detoxification, DNA-repair, stress responses, and others are playing a role in determining chemosensitivity. The use of gene expression prediction signatures have already begun to be used for the accurate prediction of treatment response in many cancers [6–10]. One such strategy developed by Lee and Theodorescu was termed the Co-Expression Extrapolation (COXEN) method [11]. It combines microarray gene expression and drug sensitivity data from a reference set to identify gene signatures that are then extrapolated to a different dataset for drug sensitivity prediction. This method has been used to successfully predict in vitro sensitivity as well as clinical outcome in bladder, breast, and non-small cell lung cancers [11–16]. It has also been implemented for *in silico* drug screening and is currently being employed in a prospective human clinical trial (NCT01228942) [11, 17].

In the recent past, clinical trials in human lung and breast cancer with similar strategies of selecting drug treatment based on gene signatures were suspended due to faulty preclinical data and improper validation of predictors, a strong reminder that proper pre-clinical validation is essential for the continued development of such methods in the clinic [18, 19]. Advanced companion animal models that share highly similar cancer genomics with humans and are routinely treated for spontaneous tumors in a clinical setting would be an ideal choice for pre-clinical validation studies.

Canine models of cancer have many advantages for translational research including a high incidence rate of spontaneous tumors that are comparable to humans both biologically and genetically [20, 21]. After the canine genome was sequenced in 2005, studies have revealed high similarity between human and canine cancers with respect to genetic homology, molecular alteration of known cancer pathways, and amplifications of known oncogenes [22–27]. Compared to rodents, larger body and tumor size in dogs allows for repeated sampling over time from the same patient and more tissue for molecular analysis [28]. Veterinary clinical trials are less expensive, can be performed in the pre-IND setting, typically represent a less pre-treated population, and due to accelerate progression of canine cancer can be completed faster than human trials [20, 21].

Canine osteosarcoma (OS) is the most common primary bone tumor in dogs and has been shown to share similar molecular alterations with the human disease [29]. Although limb sparing techniques have recently been developed for patients with existing orthopedic problems, canine OS patients are most commonly treated with amputation of the affected limb following adjuvant doxorubicin and/or carboplatin. Human and canine OS respond similarly to treatment and similar molecular pathways that are differentially expressed between poor and good responders are found in both species [30, 31].

The purpose of this study was to investigate the utility of the COXEN method in an intra- and interspecies manner between human and canine datasets, specifically in canine osteosarcoma. The reasons behind this chosen application of COXEN are three-fold: first, a successful application of COXEN across species would show the robustness of this method. Secondly, success of this study would add strength to the growing evidence that the canine cancer model can greatly impact human research, specifically in the new genomics era. Lastly, considering the wealth of human genomic and pharmacologic data available to the public, the potential to extrapolate this information into the veterinary setting through molecular comparisons of tumors would be extremely advantageous for the field of canine oncology.

## Methods

### Cell culture

A panel of 29 canine cancer cell lines used for drug screening at the Flint Animal Cancer Center (FACC) were maintained as previously described (Additional file 1: Table S1) [24]. Cell line validation was carried out by multiplex PCR on genomic DNA to confirm species of origin of each cell line as previously described [32]. Each line was additionally analyzed by short tandem repeat profiling using the Canine Stockmarks genotyping Kit (Life Technologies) per the manufacturer’s protocol and as previously described [33].

### Drug sensitivity assays

Drug sensitivity data was generated via Alamar Blue assays for cisplatin (CIS), carboplatin (CARBO), doxorubicin (DOX), and vinblastine (VBL) in the FACC panel as previously described [24], the only deviations being 48 h of drug incubation and cells were plated in 96 well plates at a density of 1500 – 5000 cells in 100 uL per well, depending on growth rate. Experiments were performed at least in triplicate, and medial dose (Dm) values were calculated.

### RNA extraction and determination of integrity

#### Canine cancer cell lines

RNA from the FACC was extracted using the Qiagen RNeasy Kit (Qiagen, Valencia, CA) according to manufacturer’s protocols. A DNase treatment step using the RNase-Free DNase Set (Qiagen, Valencia, CA) was included to ensure RNA purity.

#### Osteosarcoma tumor samples

RNA from 33 frozen tumor samples was extracted as described previously [24]. Yield and integrity for all RNA samples were calculated using a NanoDrop 1000 spectrophotometer (Thermo Scientific, Asheville, NC) and an Agilent 2100 Bioanalyzer (Agilent, Santa Clara, CA) at the Genomics and Microarray Core at the University of Colorado Denver. Clinical outcome of patients and drug treatment data was obtained from the database at the FACC. All dogs received at least one cycle of drug treatment (one cycle of each drug in the case of combination-treated dogs) before recurrence of disease in this study. Tissue collection and data acquisition is done with written owner consent and is approved through the Institutional Animal Care and Use committee (IACUC) approval number 13-4304A. As the performance of our prediction models were assessed retrospectively ethics were not required for this current study.

### Microarray gene expression analysis

RNA samples for the FACC were hybridized onto Affymetrix GeneChip Canine Genome 2.0 arrays (Affymetrix, Santa Clara, CA) and microarray analysis was performed at the Genomics and Microarray Core at the University of Colorado Denver. Raw microarray data was preprocessed with the Robust Multi-Array Average (RMA) algorithm. Information on the availability of the gene expression data for the different datasets can be found in the “Availability of Data and Materials” section of this manuscript.

### COXEN method for prediction model building

#### Standardization of data across species and microarray platforms

In addition to the normalization across samples in a dataset that occurs as part of RMA preprocessing, the gene expression data underwent within gene standardization by subtracting the mean of a probe across samples and dividing by the standard deviation. This allows genes of different mean expression intensity to be an more equal footing for the differential gene expression analysis and makes the expression data across microarray platforms more comparable to each other, especially in the case of working between human and canine datasets.

#### Differential gene expression analysis

Differentially expressed genes (DEGs) in our reference data sets were identified through either Significance Analysis of Microarrays (SAM) or *t*-test. SAM analysis with a q-value cutoff of 0.1 was used comparing the top and bottom 20 % of samples in the reference set based on drug sensitivity. T-tests were run between sensitive and resistant samples with a q-value cutoff of 0.05 to identify DEGs. If too few DEGs made the q-value cutoff, a *p*-value cutoff < 0.001 was then used.

#### Probeset matching of gene expression data

We employed four different strategies for matching probesets selected from either the Human Genome U133A or HT Human Genome U133A array to the GeneChip Canine Genome 2.0 array.

#### Best sequence homology

We used the Basic Local Alignment Search Tool (BLAST) to compare the target sequences of the human probesets with their canine orthologs and selected the match with the highest sequence homology. The blastn algorithm was selected for somewhat similar sequences, with word size = 7 and expected threshold = 100. In instances where no canine ortholog was annotated, an attempt at manual annotation was performed through sequence alignment using Affymetrix, BLAST, and USC genome browser tools. If manual annotation proved unsuccessful then the human probeset was removed from the list of candidates for COXEN analysis.

#### Best correlative match

This strategy was performed by creating correlation matrices for both human and canine probesets separately, and then a 3^rd^ correlation matrix created from each row of the first two matrices. Because the 3^rd^ matrix is created from the two original matrices, the original matrices had to be identical in dimensions. To do this, duplicates were added to the human correlation matrix to equal the number of probesets mapping to each gene on the canine matrix. The best match was selected that had the highest concordant expression with other genes in the signature across species.

#### Data collapsing by averaging method

We took our list of candidate human PSIDs and their canine orthologs and in cases where multiples existed for a given gene on either the human or canine side, the expression values were averaged. This resulted in collapsing the probeset expression data down to the gene level, reducing the data for each gene to one human and matching canine expression value. Manual annotation was attempted when no annotated canine orthologs were available.

#### Data collapsing by maximum variance method

Using the *collapseRows* function from the R package “WGCNA” [34] we collapsed the gene expression data from the probe level down to gene level before differential gene expression analysis by selecting one representative probe per gene based on maximum variance between samples, After collapsing is performed in both human and canine datasets, genes that were not present on both arrays were filtered out.

#### Selecting subset of gene signature with strong concordant expression

**Fig. 1:**
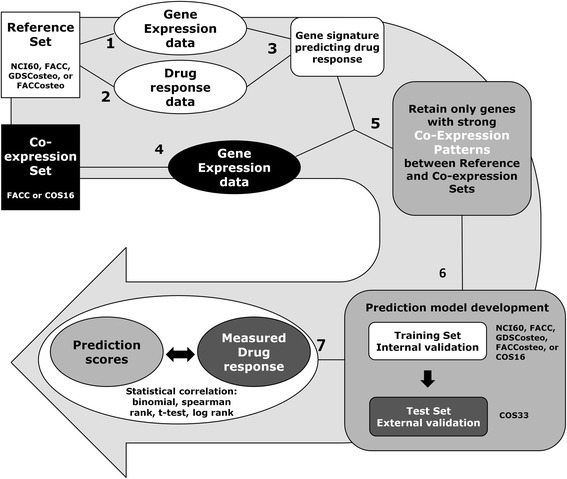
The Co-expression Extrapolation (COXEN) method. Steps 1–3 involve identifying a gene signature that predicts chemosensitivity from the reference set. Steps 4–5 involve gene expression data from the co-expression set and identifying a subset of the gene signature that shares strong co-expression with the co-expression set. The resulting genes are candidates in prediction model development in step 6, and the resulting predictions are compared to actual chemosensitivity or clinical outcome in step 7

The crucial step in the COXEN method is filtering the list of DEGs down to those that are most in “tune” with your target or test dataset, whether it is human derived DEGs that are most in tune with canine tumors, or a specific tumor type like osteosarcoma. A dataset with similar properties as your target or test set is used in this process and has been termed the “co-expression set” (Fig. 1). The selecting of co-expressed genes between the reference and co-expression datasets was based on protocols described previously by Lee et al. [11, 35]. Briefly, a correlation matrix was generated for the expression data of identified DEGs from the reference set to determine how the genes in the DEG list correlate to one another. A second correlation matrix is created with the expression data of those same DEGs pulled out from the co-expression dataset separately. Then, a 3^rd^ correlation matrix is created comparing each row of the first two matrices. Genes with correlation values higher than a cutoff (90^th^ percentile from a random null distribution or a p-value cutoff of 0.05) were selected as being strongly co-expressed between the two data sets. In other words, the genes that are retained have similar patterns of correlation with each other in both the reference and co-expression datasets. This subset of the gene signature was used for prediction model generation.

#### MiPP algorithm model generation

Prediction models were generated in Bioconductor using the Missclassification-Penalized Posteriors (MiPP) algorithm developed by Soukup, Cho and Lee [36]. Different classification methods such as linear discriminant analysis (LDA), logistic regression (LOG), linear support vector machines (SVMLIN), and radius-based function support vector machines (SVMRBF) were evaluated. Model training was performed by splitting the training set randomly several times into training and test sets, and the resultant models are then tested on more random splits of the data to determine robustness. The top 3–5 performing models are then used to predict onto a separate test dataset and the final posterior probability scores are averaged. The averaged posterior probability score has been termed the “model score”, where a value below 0.5 would be considered “resistant” to the drug and values including 0.5 and above would be considered “sensitive”. For in vivo model building, we identified candidate model genes in cell line datasets, but built the MiPP models on tumor panel data sets that were used in the co-expression step.

#### Validation of prediction models

To see how well the prediction models correlated with actual sensitivity data, we standardized both the posterior probability scores for each sample and the actual GI_50_ data by subtracting the mean and dividing by the standard deviation. A Cox proportional hazards regression correlation was then performed on the two data sets, as well as a binomial test based on random coin tossing for the testing of the models ability to successfully call a cell line “sensitive” or “resistant”. For in vivo models we plotted survival curves based on actual disease free interval data and the class predictions of “responder” or “non-responder”. Significant differences in survival curves were determined by Log Rank test.

## Results

### Human and canine cancer cell lines are similarly sensitive to chemotherapy

**Fig. 2:**
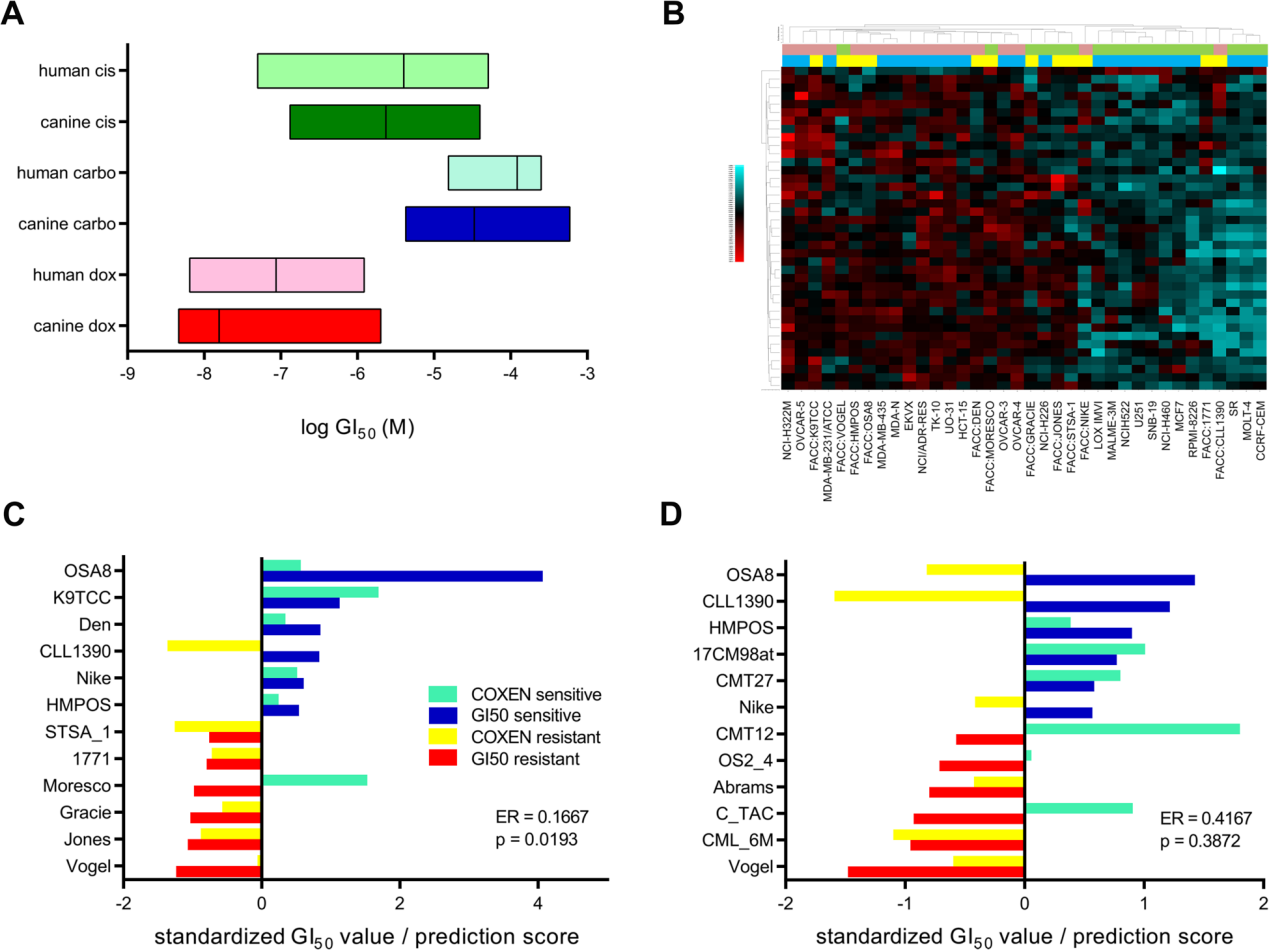
The effect of in vitro human COXEN models on predicting canine cell line sensitivity to doxorubicin or carboplatin. **a** GI_50_ ranges of the human NCI60 panel to 3 chemotherapeutics commonly used for canine osteosarcoma treatment were compared to the GI50 ranges generated in the canine FACC panel via Alamar Blue assays. **b** Differentially expressed genes based on sensitivity of NCI60 samples to doxorubicin were used in the most and least sensitive NCI60 and FACC samples in unsupervised hierarchical clustering. Light green and pink bars refer to sensitive and resistant samples, respectively. Blue and yellow bars refer to NCI60 and FACC samples, respectively. **c** & **d** COXEN predictions of doxorubicin (**c**) or carboplatin (**d**) sensitivity in the FACC cells from NCI60-trained models compared to actual GI_50_ values. Significance of accurate predictions determined by binomial test. ER = Error Rate

The two main cell line panels that were used for reference sets in our prediction model development (Fig. 1) were the human NCI60 panel and the canine FACC panel (Table 1). Since drug response and microarray gene expression data were publicly available for the NCI60 panel, we generated drug response data in the FACC panel via Alamar Blue cell viability assays for CARBO, CIS, DOX, and VBL. The range of log GI_50_ values between the human and canine panels were compared and found to be strikingly similar (Fig. 2a), although the means were significantly different between species except for with CIS (*p* = 0.0942, Mann Whitney test).

**Table 1:** Datasets used in study

Datasets	# of samples	Sample type	Tumor types represented	Microarray platform/public ID
NCI60	60	Human cancer cell lines	Breast, melanoma, central nervous system, colon, lung, leukemia, ovarian, prostate, renal	GeneChip Human Genoma U133A array (GSE5846)
GDSCosteo	10	Human osteosarcoma cell lines (subset of GDSC panel)	Osteosarcoma	GeneChip HT Human Genome U133A array (E-MTAB-783, ArrayExpress)
FACC	29	Canine cancer cell lines	Hemangiosarcoma, histiocytosis, leukemia, lymphoma, mammary tumor, mast cell, melanoma, osteosarcoma, soft tissue sarcoma, transitional cell carcinoma	GeneChip Canine Genome 2.0 array (GSE76126)
FACCosteo	10	Canine osteosarcoma cell lines (subset of FACC panel)	Osteosarcoma	GeneChip Canine Genome 2.0 array (GSE76126)
COS16	16	Canine osteosarcoma tumor samples	Osteosarcoma	GeneChip Canine Genome 2.0 array (GSE24251)
COS33	33	Canine osteosarcoma tumor samples	Osteosarcoma	GeneChip Canine Genome 2.0 array (GSE76127)

### Selecting a probeset matching strategy between the human and canine arrays

We next wanted to compare the genomic similarity of human and canine cancer cell lines in the context of drug sensitivity. RNA was extracted from untreated FACC cell lines and microarray gene expression data was generated. Thirty-nine differentially-expressed genes (DEGs) were identified in the NCI60 panel by using the 12 most and least sensitive cell lines to DOX in a SAM analysis. Since there are roughly twice as many probesets on the canine microarray chip as the human, and also on both chips there are often multiple probesets mapping to the same gene, it became apparent that a strategy for selecting the best match for the identified DEGs between species was needed. Three strategies were initially implemented and tested for matching up the probesets. The strategies named *best sequence homology, data collapsing by averaging,* and *best correlative match* are explained in full in the Supplemental Methods. Each strategy was used resulting in a human gene signature with matching canine data for both a DOX and VBL analysis. To test which strategy would produce the most predictive models, each were carried through the remaining steps of the COXEN analysis (Fig. 1). We tested a combination of all three probe matching strategies together with the five classification methods and generated prediction models. Comparing the resulting sMiPP scores and error rates associated with each model generated, *best sequence homology* was selected as an appropriate probeset matching strategy. We found multiple classification methods to be comparable in terms of overall performance and selected the commonly used LDA classification method for the remaining of our studies (Additional file 2: Table S2). During the refining process of developing models, however, we incorporated another probeset matching strategy that was automated and less time intensive than comparing the sequence homology of potential probe matches. This new method (*max variance*) involved collapsing both the human and canine array information to the gene level prior to differential gene expression analysis by selecting one probe per gene based on maximum variance between samples. It was performed using the *collapseRows* function in the “WGNCA” R package. We consider both of these methods as viable options for matching genes across platforms and used both in our attempts to optimize our prediction models.

### Human and canine cancer cell lines share comparable genomic profiles of doxorubicin sensitivity

An unsupervised hierarchical cluster analysis was performed using expression data from the 39 NCI60-derived DEGs for DOX in both sensitive and resistant NCI60 and FACC cell lines, and we observed that all the NCI60 samples separated according to DOX sensitivity (Fig. 2b). Also, 67 % (8/12) of FACC cell lines in the analysis separated according to sensitivity and interspersed with human samples, suggesting that human-derived DEGs are predictive in canine cancer cell lines (Fig. 2b). A cluster analysis was performed using NCI60-derived DEGs with the NCI60 panel and a publicly available canine osteosarcoma tumor panel (COS16, Table 1), where available samples were originally selected to determine differential gene expression between extreme groups of response to DOX (DFI < 100 days versus DFI > 300 days). The cluster analysis resulted in 92 and 100 % of the NCI60 and COS16 samples sorting by drug sensitivity, respectively (Additional file 3: Figure S1A). DEGs were then derived from the six most and least sensitive samples to DOX in the FACC panel. When cluster analysis was performed with FACC and the COS16, 100 % and 86 % of the FACC and COS16 samples sorted by drug sensitivity, respectively (Additional file 3: Figure S1B). Taken together, we’ve shown that human and canine cancer cells respond similarly to chemotherapy and that gene signatures of doxorubicin sensitivity derived from human or canine cell lines are able to separate sensitive and resistant tumor samples. These data suggest that human data can be predictive of canine data and that both human and canine cancer cell line data have predictive potential for canine osteosarcoma.

### Predictivity of human-based COXEN models on chemosensitivity in canine cancer cell lines

Before we developed models to predict clinical outcome in osteosarcoma patients, we evaluated how effective human COXEN models could predict DOX and CARBO sensitivity in canine cancer cell lines. The best NCI60 models for the prediction of FACC cells involved collapsing the microarray data to the gene level using the *max variance* method. The 12 most sensitive and resistant NCI60 lines were used as the training set and the 6 most and least sensitive cell lines in the FACC panel were used as the test set. Models with NCI60-derived genes that were co-expressed with the FACC and trained solely on the NCI60 panel were 83 % accurate in predicting DOX sensitivity in the FACC (*p* = 0.0193, binomial test) (Fig. 2c). In contrast, the NCI60-trained models were only 58 % accurate in predicting CARBO sensitivity in the FACC (*p* = 0.3872, binomial test) (Fig. 2d). Although the canine test set was involved in the co-expression step, the results of our DOX models are encouraging that interspecies prediction modeling is possible between human and canine cancer.

### Predictivity of cell line-trained COXEN models on clinical outcome in 33 independent canine osteosarcoma patients

**Fig. 3:**
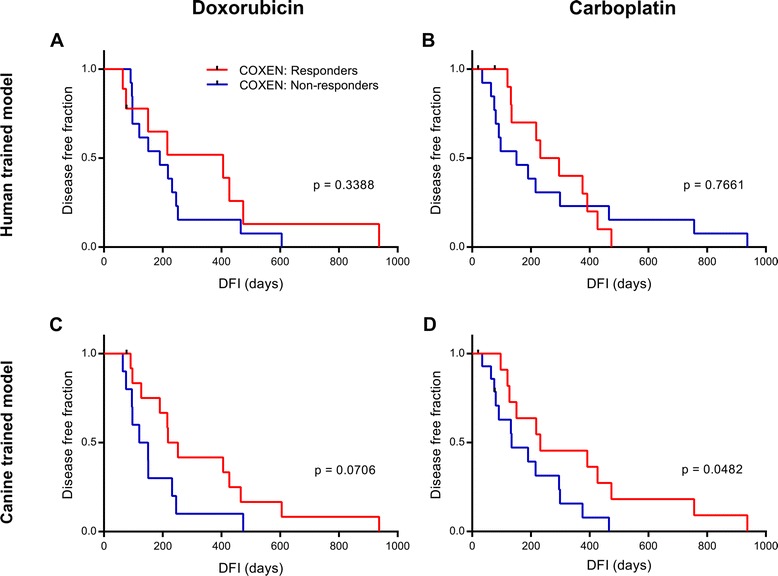
Cell line-trained models on clinical outcome in canine osteosarcoma patients treated with doxorubicin and/or carboplatin. **a** & **b** Analysis comparing the survival curves of COS33 patients predicted to respond or not respond to doxorubicin (*n* = 22) (**a**) or carboplatin (*n* = 25) (**b**) from a NCI60-trained model with the COS16 tumor panel used as the co-expression set. **c** Survival analysis of predicted responders and non-responders in the COS33 to doxorubicin from a model trained on the osteosarcoma cell line subset of the FACC panel, with the COS16 used for co-expression. **d** Survival analysis of predicted responders and non-responders in the COS33 to carboplatin from a FACC-trained model co-expressed with the COS16. Significant difference in disease free interval between predicted groups was determined by Log Rank test

Our next step was to compare the ability of cell line-trained prediction models to accurately predict clinical outcome in an independent canine osteosarcoma tumor dataset (COS33). Sample information for the COS33 tumor panel is provide in Additional file 4: Table S3. Individual reference sets used in the different models included human and canine cancer cell line panels containing multiple tumor types (NCI60, FACC) as well as osteosarcoma-only subsets from the GDSC and FACC panels (GDSCosteo, FACCosteo). After DEGs for DOX or CARBO were identified in the reference set,they were further filtered based on co-expression analysis with a canine osteosarcoma tumor panel COS16 (Table 1). Models were then trained on the corresponding reference set and tested independently on the COS33 tumor panel. Data from a historic cohort study of 470 dogs treated for osteosarcoma [37] were used to determine cutoffs between “responders” and “non-responders” in both canine osteosarcoma tumor datasets based on median disease free interval of dogs that received doxorubicin (276 days) or carboplatin (296 days). The original cutoffs for the COS16 panel reported by O’Donoghue et al. fit within our definition of “responders” and “non-responders”, so no adjustments to group classification needed to be made [31]. All of our modeling results are reported in Additional file 5: Table S4, with error rates based on external validation in the test set. The NCI60 model had an error rate of 0.3182 compared to 0.3043 for the FACC model (*p* = 0.0669 and 0.0466, binomial) (Additional file 5: Table S4). However, the NCI60 model resulted in better curve separation of predicted responders and non-responders compared to the FACC model in the survival curve analysis (Additional file 5: Table S4, Fig. 3a).

We then tested if modeling could be improved if we used osteosarcoma-only cell line panels to better reflect the test set by using the FACCosteo and GDSCosteo datasets. Interestingly, we observed that human osteosarcoma cell line model for DOX performed poorly with an error rate of 0.4348 (*p* = 0.3388), whereas the FACCosteo model for DOX was equally accurate to the FACC model with an error rate of 0.3043 (*p* = 0.0466, binomial) (Additional file 5: Table S4). Improvement of curve separation was observed in the survival curve analysis using the FACCosteo model, although only approaching significance (*p* = 0.0706, Fig. 3c).

NCI60 and FACC models of CARBO had error rates of 0.4800 and 0.3462 in predicting sensitivity in the COS33 (*p* = 0.500 and 0.0843, Additional file 5: Table S4 and Fig. 3b & d). The FACCosteo model was significantly accurate with an error rate of 0.3077 (*p* =0.0378), however in the survival curve analysis the FACC model was superior to the FACCosteo model (*p* = 0.0482 versus 0.9038, Log Rank) (Additional file 5: Table S4, Fig. 3d). A human osteosarcoma cell line model could not be performed due to absence of available CARBO sensitivity data in the GDSC panel. These data continue to suggest that CARBO models performed best when trained with canine cell line data.

### Predictivity of tumor-trained models on clinical outcome of 33 independent canine osteosarcoma patients

**Fig. 4:**
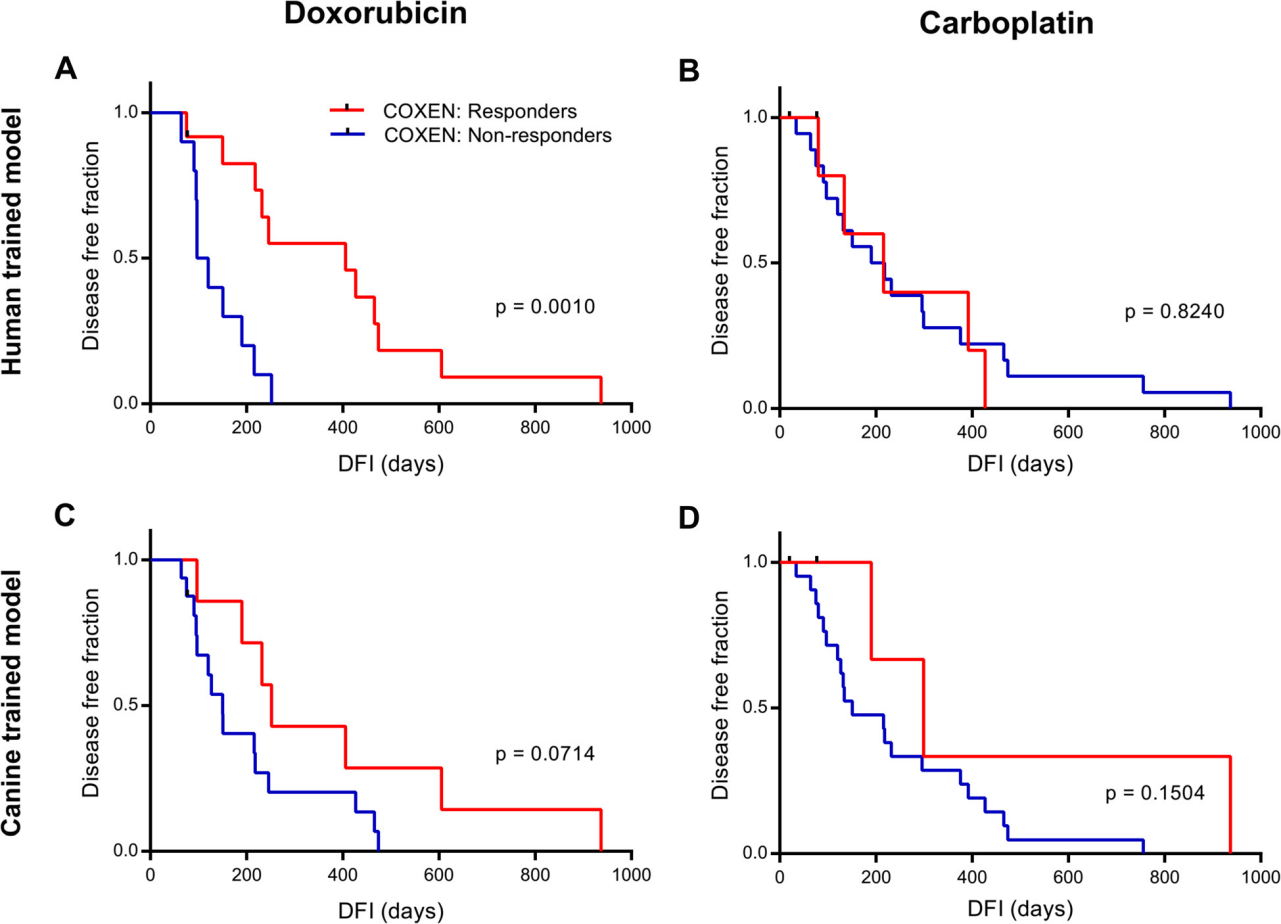
Tumor-trained models on clinical outcome in canine osteosarcoma patients treated with doxorubicin and/or carboplatin. **a** & **b** Analysis comparing the survival curves of COS33 patients predicted to respond or not respond to doxorubicin (*n* = 22) (**a**) or carboplatin (*n* = 25) (**b**) from models where genes identified from the NCI60 panel were co-expressed and trained on the COS16 tumor panel. **c** & **d** Survival analysis of predicted responders and non-responders to doxorubicin (**c**) or carboplatin (**d**) of COS33 patients from models where genes identified from the FACC panel were co-expressed and trained on the COS16 tumor panel. Significant difference in disease free interval between predicted groups was determined by Log Rank test

A study in 2010 introduced the use of “in vivo COXEN” which implemented cell line panels for the reference set but tumor panels for the model training set [14]. We tested whether using the COS16 as the model training set would improve our human and/or dog COXEN models for DOX or CARBO in the COS33. The human NCI60 DOX model that was co-expressed and trained on the COS16 performed very well with an error rate of 0.2727 (*p* = 0.0262, binomial) and a very significant separation of survival curves (*p* = 0.0010, Log Rank) (Additional file 5: Table S4, Fig. 4a). Additionally, the prediction scores significantly correlated with disease free interval in a Cox proportional hazards regression analysis with a hazard ratio of 0.03073 and a p-value of 0.00695 (Additional file 5: Table S4). In contrast, A human osteosarcoma cell line DOX model trained on the COS16 performed poorly with an error rate of 0.4783 (*p* = 0.5000, binomial) (Additional file 5: Table S4).

The canine FACC DOX model that was co-expressed and trained on the COS16 performed well but to a lesser extent with an error rate of 0.3043 (*p* = 0.0466, binomial), and a separation of survival curves that approached significance (*p* = 0.0714, Log Rank) (Additional file 5: Table S4, Fig. 4c). The FACCosteo DOX model that was trained on COS16 did not perform as well, with an error rate of 0.3913 (*p* = 0.2024) (Additional file 5: Table S4). These data show that implementing an “in vivo COXEN” method did result in our best human DOX model, and a canine DOX model that performed slightly under the best-performing FACCosteo cell line-trained model, suggesting it to be an advantageous strategy.

The human NCI60 CARBO model trained on the COS16 performed poorly with non-significant results and an error rate of 0.4000 (Additional file 5: Table S4, Fig. 4b). Both the canine FACC and FACCosteo CARBO models trained on the COS16 had significant accuracies with a shared error rate of 0.307 (*p* = 0.0378, binomial). However, none of the other tests for model performance were significant (Additional file 5: Table S4, Fig. 4d), showing that although the CARBO models were generally less accurate compared to DOX models, canine CARBO models consistently outperformed human CARBO models in our studies.

### Effect of COXEN models on clinical outcome of osteosarcoma patients receiving combination chemotherapy

The best models each for DOX and CARBO were selected based on overall performance from statistical testing (Table 2). For DOX, the NCI60 model that was co-expressed and trained on the COS16 was selected. Probeset matching between species was done using the best sequence homology method. Four genes are involved in the model: Choline kinase alpha (*CHKA*), Transducin-like enhancer of split 1 (E(sp1)homolog,Drosophila) (*TLE1*), Eukaryotic translation initiation factor 6 (*EIF6*), and Testis derived transcript (3 LIM domains) (*TES*). *CHKA* is involved in phospholipid biosynthesis and tumor cell growth. *TLE1* is a transcriptional co-repressor known to inhibit NF-kappa-B expression and WNT signaling. *EIF6* and *TES* both are involved with the cytoskeleton, *EIF6* helps link *ITGBB4* to the cytoskeleton, and *TES* is a scaffold protein that has roles in cell adhesion, cell spreading, reorganization of the actin cytoskeleton, regulates cell proliferation, and may act as a tumor suppressor (Table 2).

**Table 2:** Genes from the best COXEN models for predicting clinical response to doxorubicin and carboplatin in the COS33

Model	Gene symbol	Gene title	Function
Doxorubicin model NCI60-COS16-COS16-COS33	CHKA	Choline kinase alpha	Phospholipid biosynthesis, tumor cell growth
	TLE1	Transducin-like enhancer of split 1 (E(sp1) homolog, Drosophila)	Transcriptional co-repressor; inhibits NF-kappa-B expression and WNT signaling
	EIF6	Eukaryotic translation initiation factor 6	Helps ITGBB4 link to cytoskeleton
	TES	Testis derived transcript (3 LIM domains)	Scaffold protein; role in cell adhesion, cell spreading, reorganization of actin cytoskeleton, regulates cell proliferation, may act as a tumor suppressor
Carboplatin model FACC-COS16-FACC-COS33	OCA2	Oculocutaneous albinism II	Transporter of tyrosine within the melanocyte, may determine eye and skin color
	HES3	Hairy and enhancer of split 3 (Drosophila)	Transcriptional repressor of genes that require bHLH protein for their transcription
	LOC100688725	Uncharacterized Cytochrome C oxidase	Cytochrome C oxidase activity
	PSMD3	Proteasome (prosome, macropain) 26 s subunit, non-ATPase, 3	Involved in ATP-dependent degradation of ubiquinated proteins
	cOR4F25	cOR4F25 olfactory receptor family 4 subfamily F-like	Involved in canine olfactory system
	KIAA0922	Transmembrane protein 131-like	Integral transmembrane protein, possible involvement in immune responses

For CARBO, the FACC model that was co-expressed with the COS16 but trained back on the FACC was selected. Six genes are involved in the model: Oculocutaneous albinism II (*OCA2*), Hairy and enhancer of split 3 (Drosophila) (*HES3*), uncharacterized cytochrome C oxidase (LOC100688725), Proteasome (prosome, macropain) 26 s subunit, non-ATPase, 3 (*PSMD3*), cOR4F25 olfactory receptor family 4 subfamily F-like (*cOR4F25*), and transmembrane protein 131-like (*KIAA0922*). *OCA2* is a transporter of tyrosine within melanocytes. *HES3* is a transcriptional repressor of gene requiring bHLH protein for transcription. *LOC100688725* has cytochrome C oxidase activity. *PSMD3* is involved in ATP-dependent degradation of ubiquinated proteins. *cOR4F25* is involved in canine olfactory processes. *KIAA0922* is an integral transmembrane protein with possible involvement in immune responses (Table 2).

**Fig. 5:**
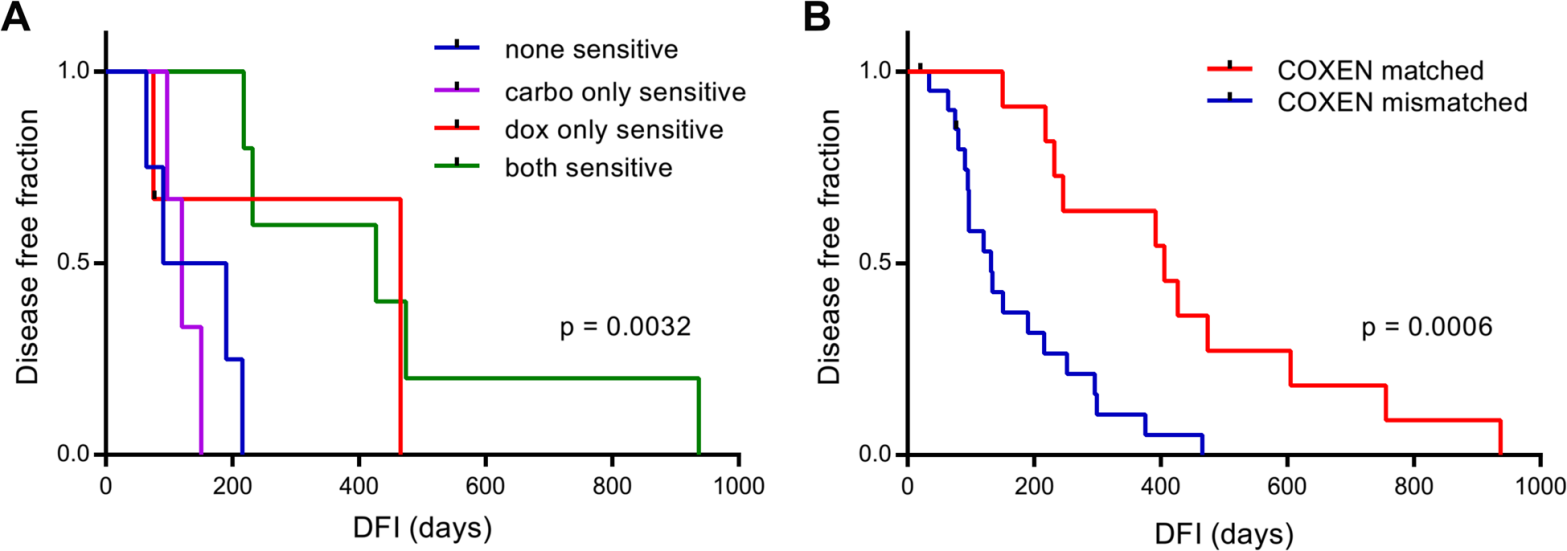
Effects of prediction-matched treatments on clinical outcome in canine osteosarcoma patients treated with doxorubicin and/or carboplatin. a Survival analysis of 15 COS33 dogs receiving doxorubicin/carboplatin combination treatment separated into four prediction groups based on the best performing doxorubicin and carboplatin models. Significant differences in curves determined by Log Rank trend test. **b** Survival analysis between COS33 dogs (*n* = 32) that were treated with a drug our best performing models predicted them to be sensitive to (“COXEN matched”) and dogs that were not (“COXEN mismatched”). For dogs that received combination treatment, a sensitive prediction on either the doxorubicin or carboplatin model was needed to be a COXEN match. Significant difference in disease free interval between the curves was determined by Log Rank test

Fifteen dogs from the COS33 panel received combination therapy of DOX and CARBO. Survival curves from dogs who received combination treatment were split into 4 groups and compared: dogs predicted to be sensitive to neither drug, dogs predicted to be sensitive to only CARBO, dogs predicted to be sensitive to only DOX, and dogs predicted to be sensitive to DOX and CARBO. In Fig. 5a a significant trend was observed between the curves, with the DOX only sensitive and DOX and CARBO sensitive groups surviving disease-free much longer than the CARBO only sensitive and resistant groups (*p* = 0.0032, Log Rank trend). These data show that dogs predicted to be sensitive to only DOX or both DOX and CARBO lived longer disease free than dogs predicted to be sensitive to CARBO or neither drug.

### Effect of COXEN-matching on clinical outcome of canine osteosarcoma patients receiving single agent or combination treatment

In order to more fully evaluate the benefit of this genomic approach of gene expression models to determine treatment in canine osteosarcoma, we classified our COS33 patients into two categories: “COXEN matched” and “COXEN mismatched”. We evaluated patient outcome if they actually received the specific treatment our COXEN models indicated. Our criteria for classification are detailed as follows: For patients treated only with DOX, if the DOX model predicted them to be sensitive (model score > 0.5), they were considered “COXEN matched”. For patients treated only with CARBO, if the CARBO model score > 0.5 they were considered “COXEN matched”. For patients receiving a combination of DOX and CARBO, both the DOX model and CARBO model score needed to be > 0.5 for them to be considered “COXEN matched”.

A survival curve analysis comparing the two groups showed that a larger percentage of COXEN matched dogs survived disease-free at all time points than COXEN mismatched dogs (*p* = 0.0006, Log Rank) (Fig. 5b). When the combination treated dogs were excluded from the analysis, COXEN matched dogs survived longer disease free than COXEN mismatched dogs (*p* = 0.0088, Log Rank) (Additional file 6: Figure S2A). Additionally, When the criteria for combination treated dogs was relaxed to consider them to be COXEN matched if they were predicted to be sensitive to at least one of the two drugs, COXEN matched dogs continued to survive longer disease free than dogs whose treatment did not match COXEN predictions (*p* = 0.003, Log Rank) (Additional file 6: Figure S2B).

We analyzed the strength of our COXEN modeling as a factor associated with DFI by performing a univariate Cox proportional hazards regression analysis with “COXEN model treatment match” as a covariate to be compared with six other clinical factors: Proximal humeral tumor location, serum alkaline phosphatase levels (ALP), monocyte count, lymphocyte count, body weight, and age at diagnosis. COXEN model treatment match was the only factor significantly associated with DFI in the univariate analysis (*p* = 0.0353) (Additional file 7: Table S5). Four of the seven factors with p-values less than 0.25 (COXEN model treatment match, age at diagnosis, body weight, and proximal humeral tumor) were than subjected to multivariate analysis followed by stepwise forwards and backwards regression modeling to determine the best subset of factors in final model. We found that COXEN model treatment match had a hazard ratio of 0.3102 which was significant (*p* = 0.0124). The other significant factor associated with DFI in our COS33 patients was body weight (hazard ratio = 1.0047, *p* = 0.0261). The last factor in the model was proximal humeral tumor location (hazard ratio = 2.3974, *p* = 0.0877). Age at diagnosis fell out of the model after stepwise regression (Table 3).

**Table 3:** Factors associated with disease free interval of COS33 patients in a multivariate analysis

Variable	HR (95 % CI)	*P* value
COXEN model treatment match	0.3102 (0.1240–0.7762)	0.0124
Body weight (continuous)	1.0047 (1.0052–1.0857)	0.0261
Proximal humeral tumor	2.3974 (0.8787–6.5407)	0.0877

*HR* hazard ratio, *CI* confidence interval, *P* values < 0.05 are in bold

Taken together, our studies suggest that gene expression models for drug sensitivity have great potential to be used in an inter- and intraspecies manner for improvement of personalized medicine for canine osteosarcoma patients.

## Discussion

The use of gene expression prediction models in personalized medicine is emerging as a potential alternative to traditional treatment strategies. In this study we were able to successfully develop prediction models for DOX or CARBO sensitivity in canine osteosarcoma using both a combination of human and canine datasets as well as canine datasets alone. The best performing DOX model was developed by first identifying DEGs from the human NCI60 panel, followed by co-expression and model training on canine osteosarcoma tumors. The ability for the COXEN method to extrapolate data from one dataset to another was able to not only be applied from an in vitro panel to an in vivo dataset, but also from one species to another. Additionally, knowing the NCI60 panel does not contain osteosarcoma cell lines makes this model even more impressive. These results have exciting implications, as it suggests that it may be feasible and beneficial to incorporate human genomic data in the development of gene expression-based predictions of treatment for canine cancer. This would be very advantageous for canine oncology, because available data needed for these types of analyses are currently much more prevalent in human research.

Although the genes in our models do not have obvious mechanisms that might influence drug sensitivity, it is interesting that *CHKA* in our DOX model has recently been shown to play a role in the sensitivity of human ovarian cancer cells to DOX, paclitaxel, and cisplatin [38]*.* Additionally, *PSMD3* from our CARBO model was identified as differentially expressed in human cancer cell lines that were sensitive or resistant to the EGFR inhibitor lapatanib [39].

In general, attempts at building prediction models for CARBO sensitivity were not as successful as they were for DOX. Additionally, models developed using only canine datasets for CARBO did consistently better than when the human NCI60 panel was incorporated in the process. A possible explanation for this was that the range of GI_50_ values in the NCI60 was the narrowest for CARBO compared to DOX and CIS, suggesting that genetic differences between “sensitive” and “resistant” groups in the CARBO data may have been minimal or were dominated by unrelated factors (Fig. 2a).

There are some limitations to this study that need to be addressed before this type of method could conceivably be implemented in the clinic. First, the relatively small sizes of our datasets made it difficult to reach statistically significant predictive power in many of our modeling iterations.. Even in instances where statistical significance was achieved as was shown with the significant trend between different prediction groups in combination-treated dogs, a sample size of 15 is not large enough for firm conclusions to be made clinically (Fig. 5a). Ideally, larger panels would serve to more fully represent the heterogeneous cancer population, leading potentially to more robust models We hope to expand the FACC panel in coming years through the establishment of new tumor-derived cell lines as well as through increased collaborations with other institutions.

Another area that needs further study is the optimization of the cutoffs between “responders” and “non-responders” in in vivo datasets. It is possible that using a different cutoff besides the median DFI in a large historical cohort may improve prediction model performance. We have recently begun a prospective study in dogs with spontaneous osteosarcoma where their chemotherapy treatment post limb amputation of either doxorubicin, carboplatin, or both will be determined based on the results of our top prediction models presented in this current study. This prospective study is planned to involve 100 dogs which will not only increase our available tumor datasets for optimizing cutoffs but will allow us the opportunity to address the issues of cost and time needed for data generation which are important for patients. We have found that the dropping price for gene expression profiling is reasonable and turnaround time of less than a week can be achieved, making this option a feasible one in the clinic. Testing our models prospectively will help to test their robustness, which we have not sufficiently done. Regardless of the limitations, our best performing models for DOX and CARBO did achieve statistically significant results in a truly independent test set, which is very encouraging.

We used the MiPP algorithm in our LDA model building process, which is designed to select the most parsimonious models without sacrificing predictive efficacy. There are advantages and disadvantages to this. Smaller models could allow for the development of gene expression-based tests similar to ones used in breast cancer diagnosis or even RTPCR protocols that would deliver quick results for patients. However, there is the opinion that prediction models with few genes tend to be less robust when tested against multiple independent datasets. As our models have only been tested in one independent test set, we cannot at present know if they would hold up to validation from additional datasets. In our investigations other model building methods besides using LDA and MiPP were employed, including principal component regression and lasso regression, which resulted in larger gene models than when we used MiPP. Unfortunately, these alternative models failed to perform as well as our best MiPP models (data not shown).

Other options for model building that might be explored in the future would be to adopt some machine learning methods with the ability to incorporate multiple types of genomic data into the modeling process. In 2014 an article reporting the results of a community-based contest of 44 competing drug sensitivity prediction algorithms concluded that although gene expression microarrays was consistently the top source for predictive power, performance could be improved with the addition of data such as methylation status of genes, copy number variation, and exome sequencing [40]. These additional types of genomic data would need to be obtained for the datasets involved in the prediction model process before these new strategies can be investigated. Ultimately, we hope that success of this strategy of personalized treatment in dogs could serve as much needed validation for similar methods in human research to move forward.

## Conclusions

This study has shown that human and canine cancer cell lines are similarly sensitive to chemotherapy and that gene expression-based modeling using canine datasets or a combination of human and canine datasets can accurately predict clinical outcome in canine osteosarcoma patients treated with adjuvant DOX and/or CARBO therapy. These results are important for human and canine cancer research for two main reasons: First, it shows the potential of an advanced animal translational model for testing genomic methods of personalized cancer treatment in a clinical setting; second, it shows the potential for canine cancer research to expand in this genomic era through the incorporation of human genomic data into their model development design, which is currently much more prevalent and available than canine datasets.

### Availability of data and materials

Gene expression data from Affymetrix Human Genome U133A arrays and U133 Plus 2.0 arrays for the human NCI-60 [11] and a panel of 16 canine osteosarcoma tumors [31] were obtained publicly from NCBI’s Gene Expression Omnibus website (dataset # GSE5846, GSE32474, and GSE24251, respectively). HT Human Genome U133A array data from the Genomics of Drug Sensitivity in Cancer (GDSC) panel was publicly available from the Array Express website (E-MTAB-783). The gene expression data for the FACC and COS33 panels were generated and made available at NCBI's Gene Expression Omnibus website (dataset # GSE76126 and GSE76127, respectively) (Table 1).

## Additional files

Additional file 1: Table S1. FACC panel info. (DOCX 17 kb) Additional file 2: Table S2. COXEN models using 5 classification methods and 3 probeset matching strategies. (DOCX 16 kb) Additional file 3: Figure S1. Human and canine cell line gene signatures for doxorubicin sensitivity accurately sort canine osteosarcoma samples. *A&B*) Differentially expressed genes based on sensitivity of NCI60 (*A*) or FACC (*B*) samples to doxorubicin were used in the most and least sensitive NCI60 or FACC samples along with COS16 tumor samples in unsupervised hierarchical clustering. Light green and pink bars refer to sensitive and resistant samples, respectively. Blue, Orange and yellow bars refer to NCI60, FACC and COS16 samples, respectively. (TIF 2026 kb) Additional file 4: Table S3. COS33 sample information. (DOCX 20 kb) Additional file 5: Table S4. COXEN modeling results for doxorubicin and carboplatin sensitivity and clinical outcome in canine datasets. (DOCX 19 kb) Additional file 6: Figure S2. Clinical outcome in canine osteosarcoma patients receiving doxorubicin and/or carboplatin whose treatments matched predictions for at least one drug. *A*) Survival analysis of 17 COS33 dogs receiving doxorubicin or carboplatin monotherapy comparing dogs whose treatment matched COXEN predictions (“COXEN matched”) with dogs whose treatment did not match (“COXEN mismatched”). *B*) Survival analysis of 32 COS33 dogs receiving doxorubicin and/or carboplatin comparing dogs whose treatment matched COXEN predictions for at least one drug (in case of combination treated dogs). Significant difference in disease free interval between the curves was determined by Log Rank test. (TIF 99560 kb) Additional file 7: Table S5. Factors associated with disease free interval of COS33 patients in a univariate analysis. (DOCX 15 kb)
