# Supplementary material for: Intra- and interspecies gene expression models for predicting drug response in canine osteosarcoma
Source: BMC Bioinformatics. 2016 Feb 19;17:93. doi: 10.1186/s12859-016-0942-8 (PMC4759767; doi:10.1186/s12859-016-0942-8)
Supplement: Additional file 1: Table S1. — FACC panel info. (DOCX 17 kb) [file 12859_2016_942_MOESM1_ESM.docx]

| **Additional file 1: Table S1. FACC panel info** | | | |
| --- | --- | --- | --- |
| **Cell Line Name** | **Tumor Type** | **Source^a^** | |
| D17 | Osteosarcoma | ATCC | |
| Abrams | Osteosarcoma | UWM | |
| Moresco | Osteosarcoma | UWM | |
| Gracie | Osteosarcoma | CSU | |
| MacKinley | Osteosarcoma | CSU | |
| Yamane | Osteosarcoma | CSU | |
| Vogel | Osteosarcoma | CSU | |
| OSA8 | Osteosarcoma | UCSF | |
| HMPOS | Osteosarcoma | Tokyo | |
| OS2.4 | Osteosarcoma | WSU | |
| 17CM98 | Melanoma | UWM | |
| CML-6M | Melanoma | AU | |
| CML-10C2 | Melanoma | AU | |
| Jones | Melanoma | CSU | |
| Parks | Melanoma | CSU | |
| CMT12 | Mammary Carcinoma | AU | |
| CMT27 | Mammary Carcinoma | AU | |
| DEN-HSA/Fitz | Hemangiosarcoma | UWM | |
| K9TCC | Bladder Carcinoma | PU | |
| Bliley | Bladder Carcinoma | CSU | |
| 1771 | Lymphoma | Upenn | |
| OSW | Lymphoma | OSU | |
| CLBL1 | Lymphoma | Aus | |
| CLL1390 | Leukemia | UCD | |
| C2 | Mast Cell | UCSF | |
| DH82 | Histiocytic Sarcoma | ATCC | |
| MH/Nike | Histiocytic Sarcoma | CSU | |
| CTAC | Thyroid Carcinoma | OSU | |
| STSA-1 | Soft-Tissue Sarcoma | UI | |
| ^a^ ATCC, American Type Culture Collection; UWM, University of Wisconsin-Madison; CSU, Colorado State University; UCSF, University of California-San Francisco; Tokyo, University of Tokyo; WSU, Washington State University; AU, Auburn University; PU, Purdue University; Upenn, University of Pennsylvania; OSU, The Ohio State University; Aus, Veterinary University of Austria; UI, University of Illinois at Urbana-Champaign. | | |  |
