# Supplementary material for: Intra- and interspecies gene expression models for predicting drug response in canine osteosarcoma
Source: BMC Bioinformatics. 2016 Feb 19;17:93. doi: 10.1186/s12859-016-0942-8 (PMC4759767; doi:10.1186/s12859-016-0942-8)
Supplement: Additional file 2: Table S2. — COXEN models using 5 classification methods and 3 probeset matching strategies. (DOCX 16 kb) [file 12859_2016_942_MOESM2_ESM.docx]

| **Additional file 2: Table S2. COXEN models using 5 classification methods and 3 probeset matching strategies** | | | | | | | |
| --- | --- | --- | --- | --- | --- | --- | --- |
| MiPP rule | drug | probeset matching strategy | candidate model genes | genes in model | sMiPP score | error rate |  |
| LDA^a^ | doxorubicin | Best homology^b^ | 8 | 1 | 0.1588 | 0.375 |  |
|  |  | Average duplicates | 9 | 4 | 0.3583 | **0.3125** |  |
|  |  | Best correlation | 15 | 11 | 0.3395 | **0.3125** |  |
|  | vinblastine | Best homology | 8 | 6 | 0.4846 | **0.25** |  |
|  |  | Average duplicates | 3 | 2 | -0.0435 | 0.5 |  |
|  |  | Best correlation | 6 | 3 | 0.1449 | 0.4375 |  |
| QDA | doxorubicin | Best homology | 8 | 3 | -0.0260 | 0.5 |  |
|  |  | Average duplicates | 9 | 8 | 0.2499 | 0.375 |  |
|  |  | Best correlation | 15 | X | X | X |  |
|  | vinblastine | Best homology | 8 | 3 | 0.3254 | **0.3125** |  |
|  |  | Average duplicates | 3 | 2 | -0.1238 | 0.5625 |  |
|  |  | Best correlation | 6 | 4 | 0.2473 | 0.375 |  |
| LOGISTIC | doxorubicin | Best homology | 8 | 2 | 0.4709 | **0.25** |  |
|  |  | Average duplicates | 9 | 3 | 0.1252 | 0.4375 |  |
|  |  | Best correlation | 15 | 1 | 0.2790 | **0.3125** |  |
|  | vinblastine | Best homology | 8 | 5 | 0.5000 | **0.25** |  |
|  |  | Average duplicates | 3 | 2 | -0.0023 | 0.5 |  |
|  |  | Best correlation | 6 | 2 | 0.2083 | 0.375 |  |
| SVMLIN | doxorubicin | Best homology | 8 | 2 | 0.1823 | **0.3125** |  |
|  |  | Average duplicates | 9 | 3 | 0.1605 | 0.4375 |  |
|  |  | Best correlation | 15 | 11 | 0.2111 | **0.3125** |  |
|  | vinblastine | Best homology | 8 | 6 | 0.3104 | **0.1875** |  |
|  |  | Average duplicates | 3 | 2 | -0.1359 | 0.5625 |  |
|  |  | Best correlation | 6 | 4 | 0.0648 | 0.4375 |  |
| SVMRBF | doxorubicin | Best homology | 8 | 3 | 0.6188 | **0.1875** |  |
|  |  | Average duplicates | 9 | 2 | 0.2475 | 0.375 |  |
|  |  | Best correlation | 15 | 3 | 0.3710 | **0.3125** |  |
|  | vinblastine | Best homology | 8 | 1 | 0.3697 | **0.3125** |  |
|  |  | Average duplicates | 3 | 2 | -0.2486 | 0.625 |  |
|  |  | Best correlation | 6 | 2 | 0.5000 | 0.5 |  |
| ^a^ LDA was selected as the best performing classification method  ^b^ ”Best homology” was selected as best performing probeset matching strategy | | | | | | | |
