# Supplementary material for: Intra- and interspecies gene expression models for predicting drug response in canine osteosarcoma
Source: BMC Bioinformatics. 2016 Feb 19;17:93. doi: 10.1186/s12859-016-0942-8 (PMC4759767; doi:10.1186/s12859-016-0942-8)
Supplement: Additional file 4: Table S3. — COS33 sample information. (DOCX 20 kb) [file 12859_2016_942_MOESM4_ESM.docx]

| **Additional file 4: Table S3. COS33 sample information** | | | | | | |
| --- | --- | --- | --- | --- | --- | --- |
| **Unique ID** | **DFI** | **Sex** | **Breed** | **Tumor Site** | **Age at Dx (years)** | **Chemotherapy treatment** |
| 146719 | 97 | MC | Mix | Femur | 8.8 | Doxorubicin |
| 160898 | 252 | FS | Mastif | Radius | 7.5 | Doxorubicin |
| 174094 | 466 | MC | Rottweiler | Radius | 6.9 | DoxCarbo |
| 194715 | 427 | MI | Old English Sheepdog | Tibia | 6.5 | DoxoCarbo |
| 195371 | 80 | MI | Rhodesian Ridgeback | Humerus | 9.4 | Carboplatin |
| 205677 | 120 | MC | Mix | Radius | 6.6 | DoxCarbo |
| 208768 | 406 | FS | Akita | Radius | 9.4 | Doxorubicin |
| 212126 | 150 | FS | Golden Retriever | Humerus | 9.4 | Doxorubicin |
| 212456 | 232 | FS | Great Dane | Humerus | 6.6 | DoxCarbo |
| 214376 | 34 | F | Great Dane | Tibia |  | Carboplatin |
| 217020 | 95 | FS | Mix | Humerus | 11.8 | Doxorubicin |
| 218959 | 474 | M | Irish Wolfhound | Radius | 5.6 | DoxCarbo |
| 219075 | 151 | MC | Mix | Femur | 7.1 | Doxorubicin |
| 219598 | 937 | MC | Australian Shepherd | Tibia | 10.4 | DoxCarbo |
| 220762 | 605 | FS | Greyhound | Femur | 7.1 | Doxorubicin |
| 221347 | 20 | MC | German Shorthaired Pointer | Radius | 9.8 | Carboplatin |
| 221562 | 218 | MC | Pyrenees | Radius | 5.1 | DoxCarbo |
| 221908 | 127 | MC | Pyrenees | Radius | 4.8 | DoxCarboCis |
| 222189 | 91 | FS | Greyhound | Humerus | 6.1 | DoxCarbo |
| 224989 | 296 | MC | Labrador Retriever | Scapula | 11.4 | Carboplatin |
| 232031 | 299 | FS | Great Dane | Radius | 8.0 | Carboplatin |
| 235194 | 246 | MC | Rottweiler | Tibia | 7.9 | Doxorubicin |
| 235468 | 64 | FS | Rottweiler | Humerus | 7.5 | DoxCarbo |
| 236226 | 190 | MC | Labrador Retriever | Radius | 8.5 | DoxCarbo |
| 237298 | 75 | MI | Leon Burger | Tibia, tallus | 5.3 | DoxCarbo |
| 239636 | 392 | FS | Mix | Tibia | 11.1 | Carboplatin |
| 239929 | 132 | FS | Saint Bernard | Radius | 4.2 | Carboplatin |
| 240749 | 216 | MC | Labrador Retriever | Tibia | 9.8 | DoxCarbo |
| 245591 | 97 | FS | Flat-coated Retriever | Radius | 8.7 | DoxCarbo |
| 252352 | 77 | MC | Labrador Retriever | Radius | 7.2 | DoxCarbo |
| 258525 | 756 | FS | Coon Hound | Radius | 8.9 | Carboplatin |
| 258688 | 376 | MC | Labrador Retriever | Humerus | 7.1 | Carboplatin |
| 260710 | 134 | FS | Labrador Retriever | Humerus | 7.7 | Carboplatin |
| DFI = disease-free interval, Dx = diagnosis, MC = castrated male, FS = spayed female, MI = intact male, M = male, F = female, “DoxCarbo”= Doxorubicin and Carboplatin, “DoxCarboCis” = Doxorubicin, Carboplatin, and Cisplatin | | | | | | |
