# Supplementary material for: Intra- and interspecies gene expression models for predicting drug response in canine osteosarcoma
Source: BMC Bioinformatics. 2016 Feb 19;17:93. doi: 10.1186/s12859-016-0942-8 (PMC4759767; doi:10.1186/s12859-016-0942-8)
Supplement: Additional file 5: Table S4. — COXEN modeling results for doxorubicin and carboplatin sensitivity and clinical outcome in canine datasets. (DOCX 19 kb) [file 12859_2016_942_MOESM5_ESM.docx]

| **Additional file 5: Table S4. COXEN modeling results for doxorubicin and carboplatin sensitivity and clinical outcome in canine datasets** | | | | | | | | | | | | | | | | |  |  |  |
| --- | --- | --- | --- | --- | --- | --- | --- | --- | --- | --- | --- | --- | --- | --- | --- | --- | --- | --- | --- |
| Reference set | | Co-express set | Train set | | Test set | | Error rate | | Binomial  p value | | Log Rank p value | | Cox Hazard Ratio | | Cox p value | | |  |  |
| **Doxorubicin models** | | | | | | | | | | | | | | | | | | | |
| **In vitro models on cell line sensitivity prediction** | | | | | | | | | | | | | | | | |  |  |  |
| NCI60 | | FACC | NCI60 | | FACC | | 0.1667^a^ | | **0.0193** | | NA | | 3.510 | | 0.214 | | |  |  |
| **In vitro models on tumor response prediction** | | | | | | | | | | | | | | | | |  |  |  |
| NCI60 | | COS16 | NCI60 | | COS33 | | 0.3182 | | 0.0669 | | 0.3388 | | 0.3498 | | 0.126 | | |  |  |
| FACC | | COS16 | FACC | | COS33 | | 0.3043 | | **0.0466** | | 0.9925 | | 0.8724 | | 0.922 | | |  |  |
| GDSCosteo | | COS16 | GDSCosteo | | COS33 | | 0.4348 | | 0.3388 | | 0.5778 | | 1.3313 | | 0.732 | | |  |  |
| FACCosteo | | COS16 | FACCosteo | | COS33 | | 0.3043 | | **0.0466** | | 0.0706 | | 0.2773 | | 0.0662 | | |  |  |
| **In vivo models on tumor response prediction** | | | | | | | | | | | | | | | | |  |  |  |
| NCI60 | | COS16 | COS16 | | COS33 | | 0.2727 | | **0.0262** | | **0.0010** | | 0.03073 | | **0.00695** | | |  |  |
| FACC | | COS16 | COS16 | | COS33 | | 0.3043 | | **0.0466** | | 0.0714 | | 0.3755 | | 0.334 | | |  |  |
| GDSCosteo | | COS16 | COS16 | | COS33 | | 0.4783 | | 0.5000 | | 0.5335 | | 0.5653 | | 0.503 | | |  |  |
| FACCosteo | | COS16 | COS16 | | COS33 | | 0.3913 | | 0.2024 | | 0.1831 | | 1.1268 | | 0.94 | | |  |  |
| **Carboplatin models** | | | | | | | | | | | | | | | | | | | |
| **In vitro models on cell line sensitivity** | | | | | | | | | | | | | | | | | | | |
| NCI60 | FACC | | | NCI60 | | FACC | | 0.4167^a^ | | 0.3872 | | NA | | 0.3799 | | 0.427 | | |  |
| **In vitro models on tumor response prediction** | | | | | | | | | | | | | | | | |  |  |  |
| NCI60 | COS16 | | | NCI60 | | COS33 | | 0.4800 | | 0.5000 | | 0.7661 | | 1.5244 | | 0.489 | | |  |
| FACC | COS16 | | | FACC | | COS33 | | 0.3462 | | 0.0843 | | **0.0482** | | 0.2894 | | 0.36 | | |  |
| GDSCosteo | COS16 | | | GDSCosteo | | COS33 | | NA*^b^* | | NA | | NA | | NA | | NA | | |  |
| FACCosteo | COS16 | | | FACCosteo | | COS33 | | 0.3077 | | **0.0378** | | 0.9038 | | 0.6326 | | 0.591 | | |  |
| **In vivo models on tumor response prediction** | | | | | | | | | | | | | | | | |  |  |  |
| NCI60 | COS16 | | | COS16 | | COS33 | | 0.4000 | | 0.2122 | | 0.8240 | | 2.0379 | | 0.556 | | |  |
| FACC | COS16 | | | COS16 | | COS33 | | 0.307 | | **0.0378** | | 0.1504 | | 0.1654 | | 0.262 | | |  |
| GDSCosteo | COS16 | | | COS16 | | COS33 | | NA*^b^* | | NA | | NA | | NA | | NA | | |  |
| FACCosteo | COS16 | | | COS16 | | COS33 | | 0.3077 | | **0.0378** | | 0.5115 | | 0.6193 | | 0.677 | | |  |
| *^a^* Error rates and tests based on a subset of most and least sensitive samples in the panel. | | | | | | | | | | | | | | | | |  |  |  |
| ***^b^*** Carboplatin was not screened in the GDSCosteo panel | | | | | | | | | | | | | | | | |  |  |  |
