# Supplementary material for: Intra- and interspecies gene expression models for predicting drug response in canine osteosarcoma
Source: BMC Bioinformatics. 2016 Feb 19;17:93. doi: 10.1186/s12859-016-0942-8 (PMC4759767; doi:10.1186/s12859-016-0942-8)
Supplement: Additional file 7: Table S5. — Factors associated with disease free interval of COS33 patients in a univariate analysis. (DOCX 15 kb) [file 12859_2016_942_MOESM7_ESM.docx]

| **Additional file 7: Table S5. Factors associated with disease free interval of COS33 patients in a univariate analysis** | | |
| --- | --- | --- |
| Variable | HR (95% CI) | *P* value |
| COXEN model treatment match | 0.4081 (0.1772-0.9400) | **0.0353*** |
| Proximal humeral tumor | 1.7370 (0.6862-4.3980) | 0.2440* |
| Alkaline phosphatase (continuous) | 0.9984 (0.9988-1.0010) | 0.7480 |
| Monocyte count (continuous) | 0.9988 (0.4957-2.0130) | 0.9970 |
| Body weight (continuous) | 0.1020 (0.9885-1.0530) | 0.2140* |
| Lymphocyte count (continuous) | 0.9208 (0.5300-1.6000) | 0.7700 |
| Age at diagnosis (continuous) | 0.8525 (0.6888-1.0550) | 0.1420* |
| HR, Hazard ratio; CI, Confidence interval; * denotes variables meeting the 0.25 cutoff to be used in multivariate analysis | | |
